# Entrapment of glucose oxidase within gold converts it to a general monosaccharide-oxidase

**DOI:** 10.1038/s41598-021-90242-2

**Published:** 2021-05-24

**Authors:** Yael Baruch-Shpigler, David Avnir

**Affiliations:** Institute of Chemistry and the Center for Nanoscience and Nanotechnology, The Hebrew University of Jerusalem, 9190401 Jerusalem, Israel

**Keywords:** Enzymes, Metals

## Abstract

We report that entrapping glucose oxidase (GOx) within metallic gold, expands its activity to become an oxidase for monosaccharides that do not have a natural enzyme with that activity—fructose and xylose—and that this entrapment also removes the enantioselectivity, rendering this enzyme capable of oxidizing the “wrong” l-enantiomer of glucose. These observations suggest that in this biomaterial adsorptive interactions of the outer regions of the protein with the gold cage, pull apart and widen the tunnel between the two monomeric units of GOx, to a degree that its stereoselectivity is compromised; then, the active sites which are more versatile than currently attributed to, are free and capable of acting on the foreign sugars. To test this proposition, we entrapped in gold l-asparaginase, which is also a dimeric enzyme (a dimer of tight dimers), and found, again, that this metallic biomaterial widens the activity of that enzyme, to include the D-amino acid counter enantiomer as well. Detailed kinetic analyses for all substrates are provided for the gold bio-composites, including determination of the difference between the activation energies towards two opposite enantiomers.

## Introduction

The classical flag of enzymes is their high specificity^1–3^. A common early picture of this specificity was the ‘lock-and-key’ visualization^4^, replaced later on with more realistic dynamic models^5,6^. Yet, not only in biochemistry but in chemistry at large, the opposite extreme is of central use: catalysts and reagents which have a broad, general spectrum of activity. In biochemistry this direction has focused on the following question: How can one generalize the activity of an enzyme beyond its evolutionary developed specific mode of action^7–9^? The importance of that branch of research is already displayed in the title of this report: Glucose oxidase (GOx) is a very common, useful, low-cost enzyme (see the highly cited "Glucose oxidase: an ideal enzyme" review^10^) of the EC 1.1.3 oxidoreductases family which utilizes oxygen for oxidation of specific CH-OH groups in various saccharides. GOx is a highly specific dimeric enzyme^11,12^, blind to most hexoses—such as the high-volume industrially important D-fructose, and to other monosaccharides such as the pentose D-xylose, a constituent of wood hemicellulose, second only to glucose in natural abundance^13^; and, it is highly enantiospecific, tailored exclusively to be active on the d-enantiomer. Furthermore, these two monosaccharaides lack oxidase enzymes of their own. Thus, expanding the enzymatic oxidative activity of GOx to other monosaccharides, is of interest especially if the saccharide in question must undergo structural changes such as the keto-enol tautomerization before being oxidized, as is the case for fructose^14^. This expansion is of interest also from the point of view of removing the enantioselectivity barrier, when such is needed, for instance in pharmaceutical relevant syntheses which utilize racemic intermediates^15^. While enzymes which have specificity for the naturally common counter enantiomer exist, for example D-amino acid oxidase^16^, enzymes which are non-enantiospecific to natural occurring enantiomers are extremely rare, an example being human deoxycytidine kinase^17,18^ that recognizes both l-ATP and d-ATP as substrates.

The main approaches for augmenting the catalytic activity of the active site of enzymes (inducing "promiscuity"^7,8^), have been based on affecting conformational changes either with or without manipulation of their amino acid building blocks. This has been achieved by a myriad of methods, mostly by moving to a non-isotropic environment—mainly adsorption^19–22^—or by enzyme engineering^23,24^ through their rational design^25^ or their directed evolution^26^. A second target—less studied than the active site—has been the tunnel leading to it. This target is attractive because in most enzymes it is stereo- and enantioselective, no less than the active site itself^27,28^. And indeed, it has been found that by adjusting or modifying the tunnel leading to the active site by enzyme engineering one can diversify, amplify and stabilize the activity of the enzyme^29^. To the best of our knowledge, the use of adsorptive interactions to change the conformation of the tunnel, has not yet been reported—as proposed below, this is most probably the key reason for our reported expansion of GOx activity.

**Figure 1:**
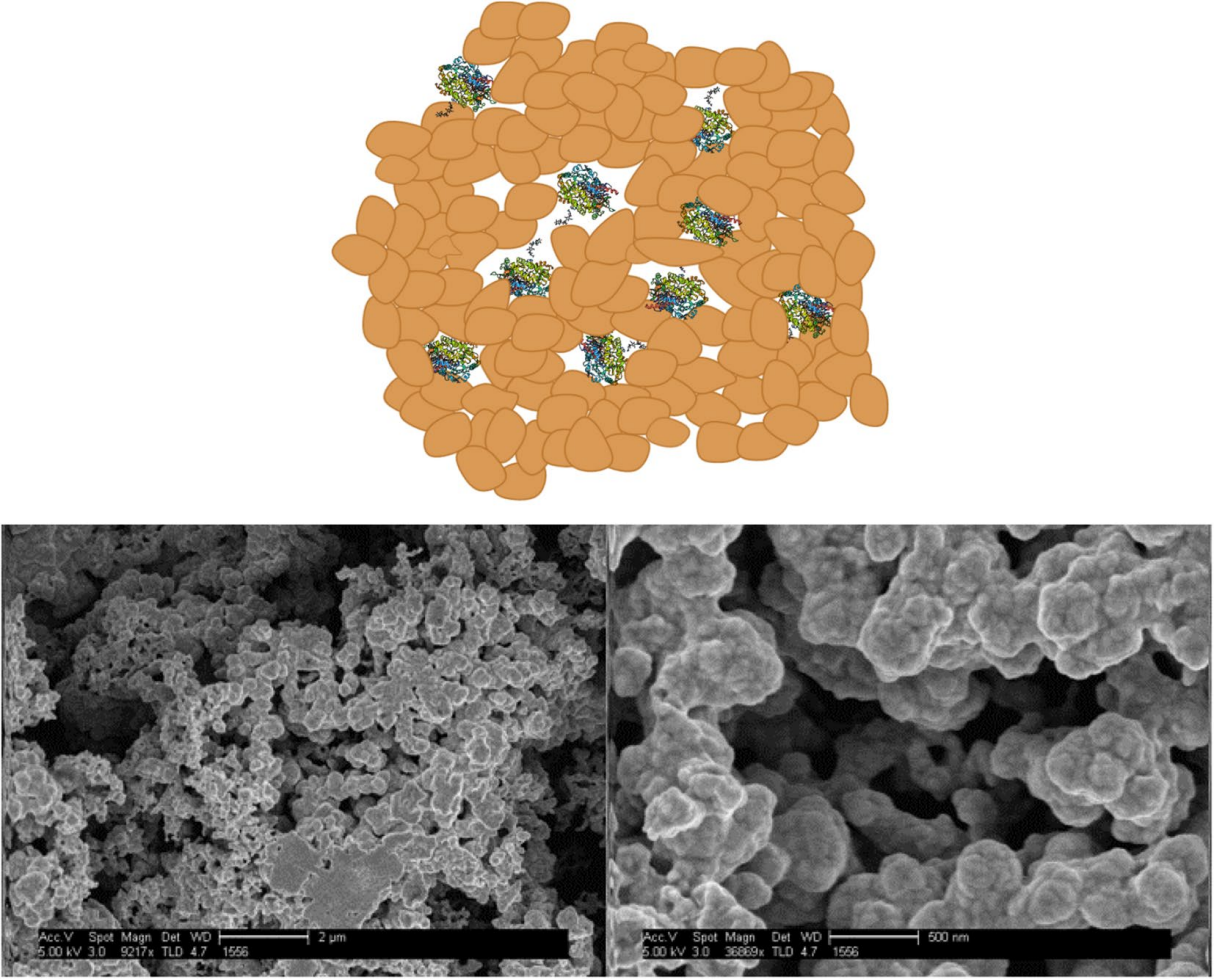
Top: Illustration of the morphology of the three-dimensional gold cages within which the enzyme molecules reside (not to scale). Bottom: HR-SEM images of glucose-oxidase@Au (left: bar = 2 µm, right: bar = 500 nm).

Here we report that entrapment of GOx within metallic gold, expands its oxidase activity to diverse monosaccharides which include, as already mentioned above, another hexose–fructose, a pentose–xylose and the counter enantiomer l-glucose. Briefly, the resulting enzyme@gold is a porous tightly aggregated metallic matrix (Fig. 1) within which the enzyme molecules are encaged, and yet are accessible through the pores network to incoming substrate molecules, and open for product molecules to diffuse out. The enzyme molecules are held inside the gold cages by a combination of physical inability to escape and interactions of gold-complexing moieties of amino acids (SH, NH, COOH) at the outer regions of the protein surface with the metallic surface around it inside the cages (Fig. 1). It turned out that the metallic matrix has excellent protein protecting capabilities, helping the enzyme to endure non-favorable conditions, such as extreme pH values and high temperatures^28^. It should be emphasized that these features reflect the fact that the entrapment results in a three-dimensional (3D) architecture, completely different^30^ from the commonly used two-dimensional (2D) physical or chemical immobilization methods to exposed surfaces (particles, electrodes, etc.). While in 2D immobilizations only one moiety or side of the protein is in contact with the immobilizing surface leaving the molecule exposed to its surrounding environment, 3D entrapment encompasses the molecule from all its sides inside the cages, utilizing the amino acids functional groups mentioned above for tight fixation with the metallic matrix. As shown elsewhere^30^, this leads to significant enhancement of thermal stability, of stability against harsh chemicals, and of the inability to be washed away. These unique features of the 3D entrapment led us to consider the possibility that the strong interactions of the entrapped enzyme with the metal, may lead to induced conformational changes that could affect the activity of the enzyme; and indeed, we report here that this is the case. Finally, since, as mentioned above, the ability of an enzyme to act on the "wrong" enantiomer is of interest in pharmaceutical syntheses^30,31^, we checked whether the entrapment within gold might alter other enzymes to become non-enantioselective. Indeed, we found that gold entrapped asparaginase (selected because of the use of this enzyme as a treatment of acute leukemia^32^, and because of its dimeric structure^33^—see below) reacts with d-asparagine almost as efficiently as with natural l-enantiomer, thus hinting to a potential generality of the ability of enriching enzymes scope of activity by entrapment in gold.

## Results and discussion

**Figure 2:**
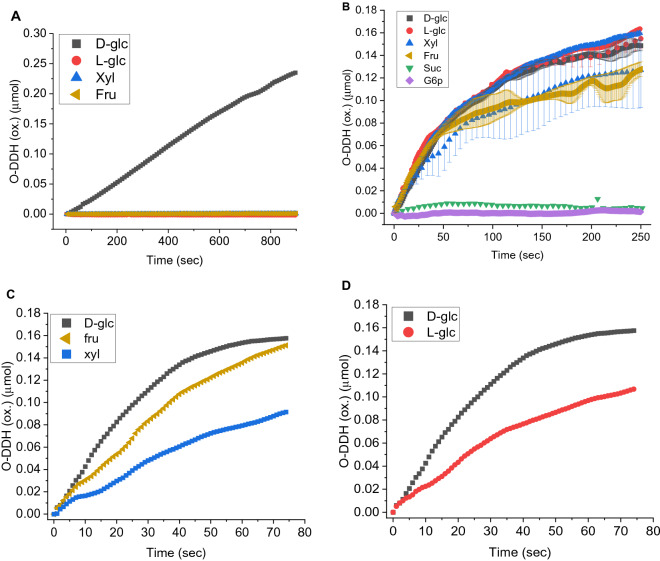
(**A**) The lack of activity of free GOx in solution towards fructose, xylose, and l-glucose (overlapping lines at the bottom), compared to the activity towards d-glucose^34^. (**B**) The activity of GOx@Au towards fructose, xylose and l-glucose and d-glucose (under saturation conditions of sugars concentration of 89.5 mM). Typical error bars of each of the monosaccharides are indicated in this figure, using the same color code. (**C**) The activities of GOx@Au towards fructose and xylose at non-saturation sugar concentrations of 15 mM and 12.5 mM, respectively. (**D**) The activity of GOx@Au towards the two enantiomers of glucose (non-saturation sugar concentrations of 15 mM).

We begin with the observations: It can be seen in the HR-SEM images (Fig. 1) that the resulting material is hierarchically porous. The composites are comprised of elementary gold nanocrystals, that are aggregated further and further to create the larger clusters. The activity of free and gold-entrapped GOx on fructose, xylose and l-glucose in comparison to d-glucose is shown in Fig. 2. First, it confirms the total lack of activity of the free enzyme on the three GOx non-native monosaccharides (Fig. 2A). However, under saturation conditions, the activity of GOx@Au towards these three "alien" saccharides is high, nearly coinciding with each other and with d-glucose (Fig. 2B). Differences are magnified when lower concentrations are taken, such as needed for the Michaelis–Menten kinetics analysis, and these are shown Fig. 2C, D.

**Figure 3:**
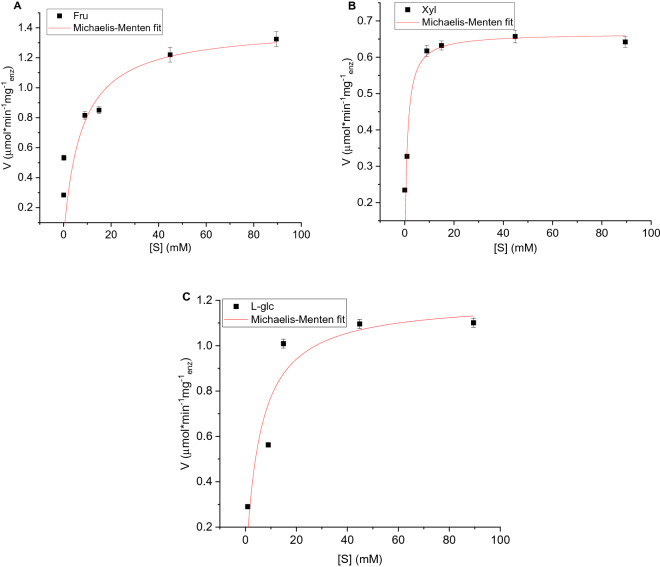
Michaelis–Menten analyses of GOx@Au activity towards (**A**) fructose, (**B**) xylose, and (**C**) l-glucose; error bars are shown.

These kinetic profiles show compliance with the Michaelis–Menten model (Fig. 3), and the resulting apparent K_m_ and V_max_ values are collected in Table 1. It was also important to establish what is the limit of this observation—it turned out that bulkier saccharides—sucrose and glucose-6-phosphate—cannot penetrate and reach the active site (Fig. 1B; the latter could also be influenced by the charge on the phosphate group). These observations—the similar kinetics at high concentrations, the lower but still significant activities observed at the lower concentration, the K_m_ and V_max_ values, the fact that the active site of the entrapped enzyme is insensitive to the handedness of the sugar molecule, and the upper size limit, are discussed below.

**Table 1:** Michaelis–Menten analysis parameters.

Enzyme@gold	Substrate	K_M_ (mM)	V_max_ (U mg^−1^)
Glucose oxidase	d-glucose	0.5	1.8
Glucose oxidase	Fructose	7.3	1.4
Glucose oxidase	Xylose	0.9	0.7
Glucose oxidase	l-glucose	5.5	1.2
Asparaginase	l-asparagine	0.35	0.56
Asparaginase	d-asparagine	0.35	0.54

**Figure 4:**
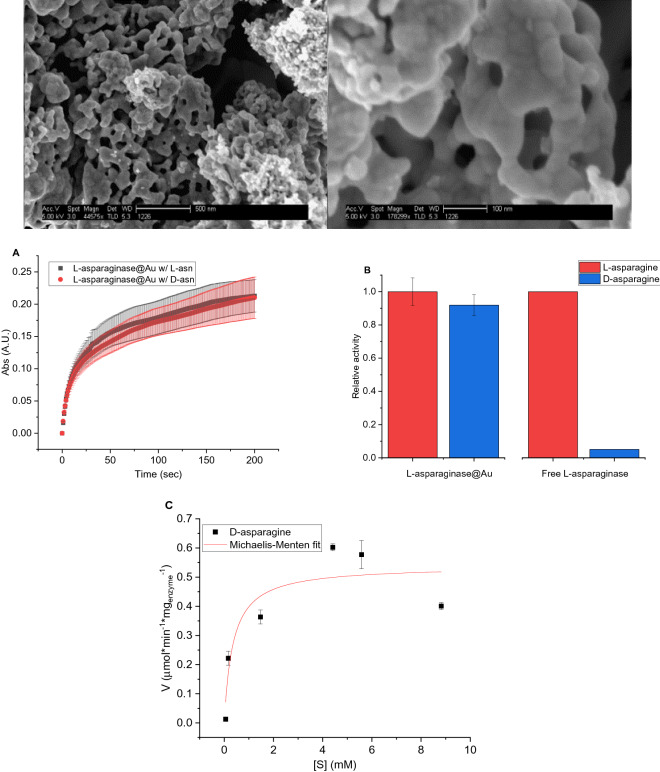
Top: HR-SEM images of l-asparaginase@Au (bars: left = 500 nm, right = 100 nm). (**A**) The activity of l-asparaginase@Au towards the two enantiomers of asparagine (saturation conditions). The error bars are indicated as in Fig. 2. (**B**) The relative initial velocities of l-asparaginase@Au (left) and of free l-asparaginase (right) towards l-asparagine (red) and d-asparagine (blue) at saturation concentrations, (**C**) Michaelis–Menten analysis of the activity of l-asparaginase@Au towards d-asparagine, with error bars based on the initial velocities.

The loss of enantioselectivity of entrapped GOx is interesting, not only because of the reasons given in the introduction, but also because it points, as we shall propose below, to the possibility that the origin of the enantioselectivity of native GOx is the tunnel rather than the active site itself. This re-focusing on the origin of the enzyme enantioselectivity prompted us to see if similar observations may be found in other enzymes. For that purpose, we have selected asparaginase, which is a dimer of two tightly held pairs of monomers^33^. This enzyme was entrapped by a similar procedure to that of GOx entrapment, resulting in a similar hierarchical porous structure, seen in the HR-SEM images of Fig. 4-top. The results for l-asparaginase@Au are shown in Figs. 4A, B: These clearly show that the entrapped enzyme is active towards d-asparagine almost as towards its primary substrate, l-asparagine. Almost no activity of the free enzyme towards l-asparagine is observed (Fig. 4B). Michaelis–Menten analysis reveals approximate compliance with that equation, and yet it allows the comparative analysis of the activity of the entrapped enzyme on both l- and d-asparagine (Fig. 4C, Table 1) showing that the entrapped enzyme acts on both enantiomers quite similarly.

**Figure 5:**
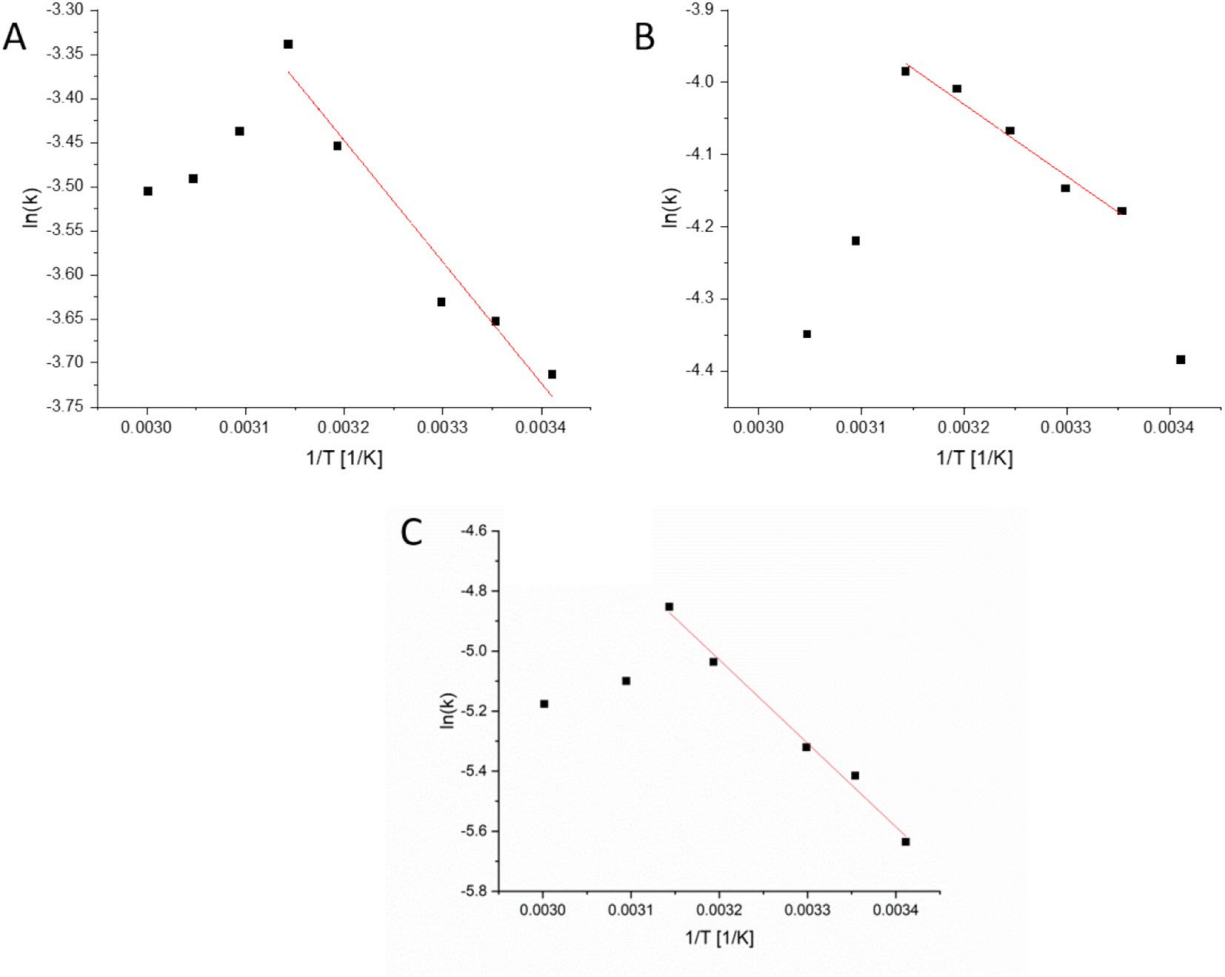
Arrhenius activation energy analysis of (**A**) free l-asparaginase towards l-asparagine, (**B**) l-asparaginase@Au towards l-asparagine, and (**C**) l-asparaginase@Au towards d-asparagine.

This enzyme allows the determination of the apparent activation energies for the two opposing enantiomers (in GOx this analysis is problematic because the activity of the indicating peroxidase enzyme is affected by temperature changes as well). The results of the Arrhenius analyses are shown in Fig. 5. Note that the Arrhenius plots show two domains—a negative slope domain, and a positive slope domain at the higher temperatures. The activation energy for the enzymatic process is taken, as usual, from the negative slope domain, a domain where increase in temperature accelerates the reaction. However, beyond a critical temperature (45 °C for all three cases), thermal denaturing governs, and the activity decreases. For l-asparagine the activation energy required by the entrapped enzyme is 8.29 kJ/mol (somewhat lower than that of the free enzyme—11.45 kJ/mol (12.07 kJ/mol in ref.^35^). For d-asparagine it is higher—23.1 kJ/mol—reflecting the fact that the enzyme finds it more difficult to process the "wrong" enantiomer; yet the significant observation that at room temperature it is able to perform the reaction at all.

**Figure 6:**
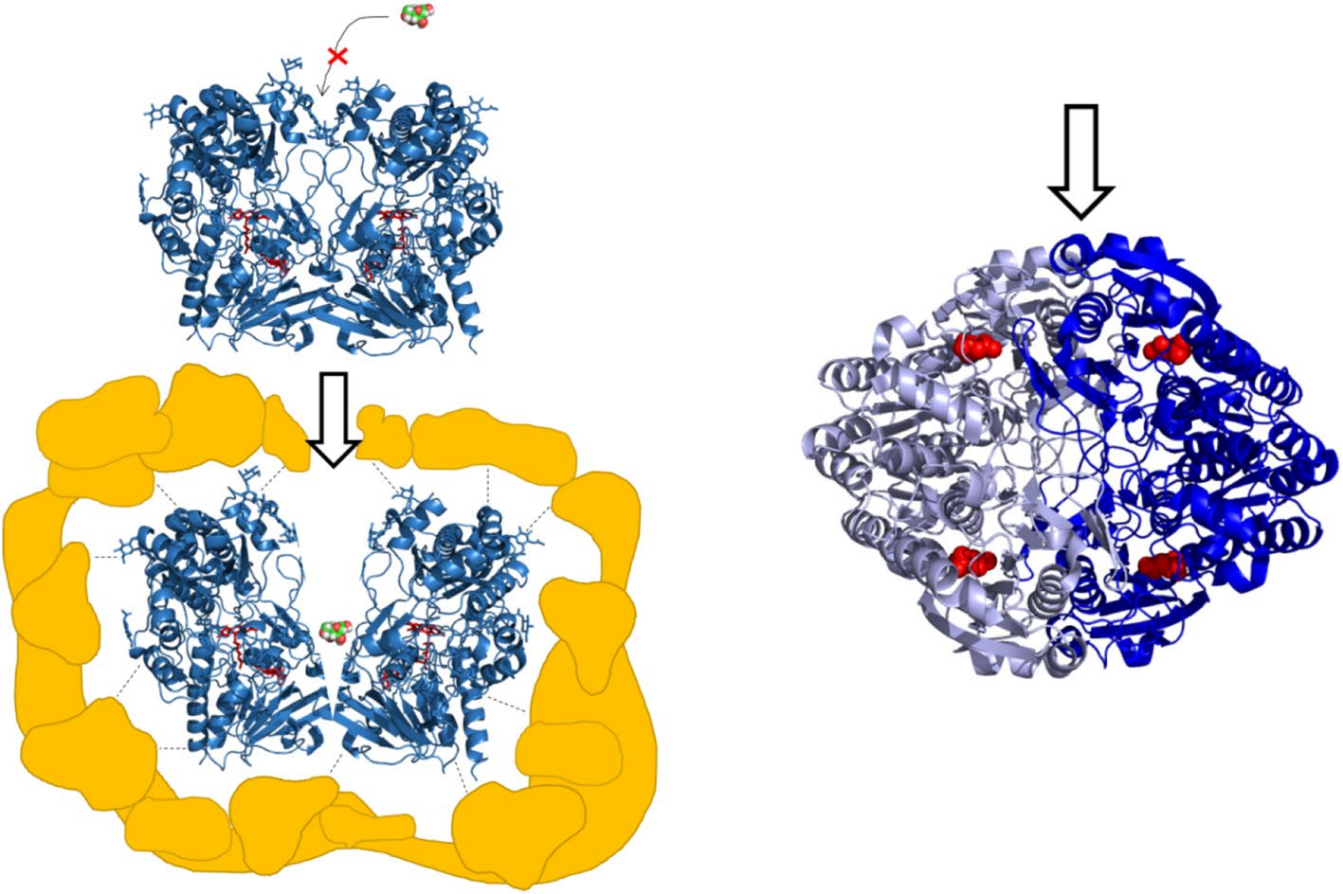
Suggested mechanism. Top left: Free GOx—fructose cannot enter the tunnel to reach active site. Bottom left: The interactions of the monomeric units of GOx with the gold, affect some separation of the monomeric units (exaggerated in the scheme for explanatory purposes), widening the tunnel, and allowing fructose to enter the relatively non-altered pair of active sites. (RCSB ID: 1GPE) Right: Asparaginase is a dimer of two pairs—colored in blue and silver—of tightly held dimers. Interactions of the outer surface as shown for GOx are similarly capable of opening the interface between the silver and blue dimers, easing the entrance (top arrow) of the “wrong” d-asparagine. (RCSB ID: 6EOK).

What then makes gold such a special modifying immobilization matrix, and what is the interpretation of these observations? As already mentioned in the introduction, gold is unique in that it interacts strongly with many of the amino acid functional groups at the outer, exposed zones of the protein. These include the well-known interactions with the SH group of cysteine^36,37^ and its oxidized cystine S–S group^38^, with amino acids carrying the CH–NH group (arginine, histidine, tryptophan)^39–41^, with the carboxylates^42^ of aspartic and glutamic acids that govern the overall negatively charged GOx, and through pi-interactions with the aromatic rings of phenylalanine and tyrosine^43^. So, we have a dopant which strongly interacts with its environment by a host of Au adsorptive bonds, which must affect the conformation of the protein, and indeed, conformational changes induced in enzymes by adsorption on gold, are well documented^44,45^, including GOx^46,47^. Next, we need to consider what are the conformational changes in GOx that could lead to the observations of broadening the scope of activity and losing the enantioselectivity? For that purpose, let us have a closer look at the GOx' structure: GOx appears in oligomeric forms, and the main active one is a dimer of two monomeric units (**C**_**2**_-symmetry) held together by non-covalent bonds (Fig. 6). The two active sites are accessed down a tunnel formed at the interface between the two monomeric units and are located to the "left" and "right" of the interface wall^48^. Since the two units are held by polar interactions which are weaker than covalent links, we propose that a main conformational change that the gold-entrapped enzyme undergoes, is a spreading apparat of the two units, opening it up to a level where the tunnel's d-glucose specificity is lost, and other sugar-molecules of approximately the same size can penetrate and reach the active sites. From the fact that the active sites can still process the native d-glucose, we can learn that conformational changes in the active site, if at all, are small to the degree that the mutual orientation of the amino-acids and cofactors needed for the catalysis, are not shifted greatly (unlike the pulling apparat the more loosely held monomers). But an even more significant conclusion can be reached from the fact that the active sites can process an additional hexose, a pentose, and the counter enantiomer, and that is that the stereochemical restriction of what is linked to the CH–OH moiety on which this enzyme operates, is weak: The active site overcomes structural variations in the sugar molecule that carries that moiety. All of which leads us to propose that the stereospecificity and enantiospecificity of free GOx are due mainly to action of the tunnel as a "gate-keeper" and not to the stereo-restrictions of the active site. The numbers behind this qualitative picture are the Michaelis–Menten parameters, which do show lower affinity (higher K_m_ values, lower V_max_ values) of the "alien sugars", but still vivid activity. The steric limit, as mentioned above, is that for larger sugar molecules such as the disaccharide sucrose, the penetration to the active site is still unattainable. The key role of the tunnel is also seen by the observation that under saturation conditions the enzymatic reactions rates for all sugar molecules almost coincide—that is, the rate limiting step is the entrance to the tunnel which under high concentration conditions is partially blocked by the over-crowding at that zone: but once the sugar molecule enters, it is transported relatively freely to the active sites.

Interestingly, asparaginase carries structural features which are similar to GOx from the point of view of interpreting the ability of the entrapped enzyme to process both enantiomers: Asparaginase is a homotetrameric protein^49^ composed of a pair of strongly held dimers (termed intimate^50^ or tight^51^ dimers) with two pairs of active sites, accessible through the interface between these tight dimers (Fig. 6, right). So, again, we are in a situation of interactions of the overall negatively charged asparaginase, with the surface of the gold cage, which loosen the entrance to the active sites; and, again, the ability to operate on the “wrong” enantiomer, points to a situation in which the specificity in solution is mainly due to the restriction of the entrance tunnel, and not of the active site itself. This similarity of GOx and asparaginase seems to suggest that the importance of the access to the active site as the stereochemical filter should be at focus when considering the specificity of enzymes, no less—in some cases perhaps even more—than the stereochemistry of the active site itself.

## In conclusion

The ability to use the well-known, well-characterized, inexpensive oxidase of d-glucose as a more general monosaccharide-oxidase, opens the possibility to extend this enzyme’s many applications to new directions, such as using a single enzyme for sugar mixtures, rather than multiple-enzymatic systems for the construction of a biofuel cell^52^. Moreover, GOx can now be utilized as an enzyme for monosaccharides that otherwise do not have naturally occurring oxidase enzymes, such as fructose and xylose. Furthermore, the generality of this phenomenon, exemplified by the extending the activity of l-asparagine to the counter d-enantiomer, indicates that this entrapment method could be used to diversify the substrate range of many types of enzymes. The observations made in this report highlight on one hand the importance of the entrance tunnel of enzymes to their active site as a stereoselective gate keeper, and on the other hand indicate that active sites are capable of operating on more than allowed by the gatekeeper. We comment that these observations and their interpretation agree with the notion that promiscuity characterizes early evolutionary stages leading to, over the eons, highly specialized activity^53^. That is, first evolved the catalytic sites, tailored more towards activity on specific functional groups and less towards their stereochemistry, and for which oligomeric length-scales are enough; and then, later-on, specificity was introduced by means of growing into larger proteins, that needed to create the selective tunnel of entry to the active site.

## Experimental details

### Chemicals, enzymes, and reagents

Sodium tetrachloroaurate(iii) dihydrate was purchased from Alfa Aesar. Zinc (granular, 20–30 mesh, ACS reagent, ≥ 99.8%) was purchased from Sigma Aldrich. *Enzymes, their substrates, and analytical reagents*: glucose oxidase from *A. niger* (135 U mg^−1^) and *o*-dianisidine were purchased from Sigma Aldrich. d-glucose was purchased from Honeywell Riedel-de Haën Research Chemicals. Glucose-6-phosphate, l-glucose and sucrose were purchased from Sigma Aldrich. Fructose and xylose were purchased from Alfa Aesar. Asparaginase from *E. coli* (∼250 U mg^−1^) was purchased from Prospec Chemicals. l-asparagine was purchased from Sigma Aldrich and d-asparagine was purchased from Alfa Aesar. Nessler's reagent (K_2_HgI_4_) was purchased from Fisher Chemical. Peroxidase from horseradish (∼200 U mg^−1^) was purchased from Sigma Aldrich. *Buffers (all of the weight values are for a volume of 500 mL of buffer)*: 50 mM sodium acetate buffer (pH = 5.1, 35 °C) was prepared for glucose oxidase by dissolving 2.05 g of sodium acetate anhydrous in distilled water, adjusting the pH to 5.1 with 1 M HCl solution. 50 mM Tris–HCl buffer (pH 8.6, 37 °C) was prepared for asparaginase by dissolving 3.03 g of tris(hydroxymethyl)aminomethane in distilled water and adjusting the pH to 8.6 with 1 M HCl solution.

### Enzyme entrapment procedures

These were described in detail in ref.^34^. Very briefly, 161.5 mg of the gold salt was dissolved in 3.49 mL water to which 4.10 mL of 50 mM sodium acetate buffer (pH 5.1) was added. The pH was then adjusted to neutral with NaOH and 1.0 mL solution of GOx (0.1 mg/mL, 135 U/mg, in buffer) was added. Finally, 40 mg of zinc granules were added and the mixture was stirred at room temperature overnight. The resulting GOx@Au precipitate was filtered, washed and dried, yielding ~ 115 mg of GOx@Au.

For the entrapment of l-asparaginase@Au, the enzyme solution was of 0.5 mg/mL, ~ 250 U/mg in TDW and the buffer solution used in the entrapment procedure was 50 mM Tris–HCl buffer in pH = 8.6. In order to make sure that all of the enzyme was entrapped, the supernatant solution was tested for residual activity.

### Bioactivity assays

#### Oxidation of fructose with GOx@Au

The composite gold powder prepared above (∼115 mg) was placed in a polystyrene cuvette. Next, the following reagents were added to the cuvette: 0.1 mL of horseradish peroxidase containing ∼200 U mg^−1^ (0.3 mg mL^−1^) in TDW, 0.1 mL of 50 mM sodium acetate buffer solution and a mixture of 2.4 mL of 0.21 mM *o*-dianisidine solution and 0.5 mL of 10% (w/v) fructose solution (89.5 mM, a saturation concentration) containing 0.28 mmoles of the sugar. The enzymatic activity was measured spectrophotometrically by following the increase in absorbance at 500 nm, at 35 °C. Comparative measurements in solution were carried out by a standard procedure^54^, where the only change was the use of fructose instead of d-glucose.

#### Oxidations of xylose and l-glucose by GOx@Au

These were carried out similarly with solutions of 89.5 mM l-glucose (0.28 mmoles) or 107 mM of xylose (0.33 mmoles). The free enzyme solutions were tested by the same standard procedure.

#### l-asparaginase@Au activity on d-asparagine

The composite gold powder prepared above (∼115 mg) was placed in a polystyrene cuvette. Next, the following reagents were added to the cuvette: 1.38 mL of 50 mM Tris–HCl buffer solution, 1.18 mL TDW, 0.3 mL Nessler's solution and 0.14 mL of 189 mM d-asparagine solution containing 0.026 mmoles of the D-amino acid. The enzymatic activity was measured spectrophotometrically by following the increase in absorbance at 436 nm, 37 °C. As shown in ref.^34^, entrapped asparaginase—but not the free one—is protected by the gold matrix from the extreme alkaline pH of Nessler reagent. The activity assay of the free d-asparagine was therefore conducted differently, as described in ref.^34^ for the l-enantiomer. The influence of zinc on the activity of the free enzyme was checked, to ensure that the presence of zinc did not interrupt the successful entrapment and activity of the enzymes.

#### Additional bioactivity characterizations

For Michaelis–Menten analysis, the initial reaction velocities (V_0_), marked by the initial slope of the plot, for GOx@Au were measured with the various sugars (fructose, xylose and l-glucose) at concentrations ranging between 1.79*10^−3^ mM and 89.5 mM. The activation energy for l-asparaginase@Au was obtained by conducting the enzymatic assay at the saturation concentration of 189 mM at temperatures ranging between 20 and 60 °C. The activation energy of free asparaginase was conducted in the same manner with the free enzyme is assay. The data are normalized per mg enzyme that was initially added to the entrapment procedure, optimized to full entrapment without leaking, as determined from supernatant analysis. In the dose–response/Michaelis–Menten kinetic analyses—the error for each initial velocity (initial slope) was used. Control experiment in which gold was tested for catalytic activity was reported in ref.^30^, and showed no activity on its own. Other control experiments on inactivation are describes in ref.^30^ as well—in solution the enzymes lose their activity, while immobilization in gold retains their activity.

### Material characterization

UV–Visible (UV–Vis) absorption spectroscopy was carried out using JASCO V-630 spectrophotometer. High-resolution scanning electron microscopy (HR-SEM) analysis was carried out on a Sirion (FEI) high-resolution SEM instrument. The protein structures were taken from the RCSB protein data bank.

## Data Availability

The raw/processed data required to reproduce these findings cannot be shared at this time due to technical or time limitations.
